# New evidence for the therapeutic potential of curcumin to treat nonalcoholic fatty liver disease in humans

**DOI:** 10.1371/journal.pone.0172900

**Published:** 2017-03-03

**Authors:** María Eugenia Inzaugarat, Elena De Matteo, Placida Baz, Diego Lucero, Cecilia Claudia García, Esteban Gonzalez Ballerga, Jorge Daruich, Juan Antonio Sorda, Miriam Ruth Wald, Alejandra Claudia Cherñavsky

**Affiliations:** 1 Instituto de Inmunología, Genética y Metabolismo-CONICET- Universidad de Buenos Aires, Buenos Aires, Argentina; 2 Hospital de Niños “Dr. R. Gutiérrez”, Servicio de Patología, Buenos Aires, Argentina; 3 Universidad de Buenos Aires, Facultad de Farmacia y Bioquímica - Departamento de Bioquímica Clínica, Laboratorio de Lípidos y Arterioesclerosis, Buenos Aires, Argentina; 4 Universidad de Buenos Aires, Hospital de Clínicas "José de San Martin"- División de Gastroenterología, Buenos Aires, Argentina; 5 Instituto de Investigaciones Biomédicas (BIOMED)- Universidad católica Argentina-Consejo Nacional de Investigaciones Científicas y Tecnológicas, Buenos Aires, Argentina; Alabama State University, UNITED STATES

## Abstract

**Introduction:**

The immune system acts on different metabolic tissues that are implicated in the pathogenesis of nonalcoholic fatty liver disease (NAFLD). Leptin and linoleic acid have the ability to potentially affect immune cells, whereas curcumin is a known natural polyphenol with antioxidant and anti-inflammatory properties.

**Aims:**

This study was designed to evaluate the pro-inflammatory and pro-oxidant effects of leptin and linoleic acid on immune cells from patients with NAFLD and to corroborate the modulatory effects of curcumin and its preventive properties against the progression of NAFLD using a high-fat diet (HFD)-induced NAFLD/nonalcoholic steatohepatitis mouse model.

**Results:**

The ex vivo experiments showed that linoleic acid increased the production of reactive oxygen species in monocytes and liver macrophages, whereas leptin enhanced tumor necrosis factor-α (TNF-α) production in monocytes and interferon-γ production in circulating CD4^+^ cells. Conversely, oral administration of curcumin prevented HFD-induced liver injury, metabolic alterations, intrahepatic CD4^+^ cell accumulation and the linoleic acid- and leptin- induced pro-inflammatory and pro-oxidant effects on mouse liver macrophages.

**Conclusion:**

Our findings provide new evidence for the therapeutic potential of curcumin to treat human NAFLD. However, the development of a preventive treatment targeting human circulating monocytes and liver macrophages as well as peripheral and hepatic CD4^+^ cells requires additional research.

## Introduction

The spectrum of nonalcoholic fatty liver disease (NAFLD) ranges from simple steatosis to nonalcoholic steatohepatitis, liver fibrosis, cirrhosis, and hepatocellular carcinoma [1]. Many dysregulated factors involved in NAFLD act in parallel, particularly gut-derived and adipose tissue factors, to finally result in liver inflammation [2]. Kupffer cell activation plays a central role in NAFLD progression through the production of pro-inflammatory cytokines and type 1 interferon (IFN), the promotion of leukocyte infiltration, and fibrogenesis [3]. When baseline inflammation is present, insulin-resistant hepatocytes increase cysteine-cysteine motif chemokine ligand 20 (CCL20) expression, which subsequently recruits lymphocytes into the liver [4].

The immune system plays roles in the metabolic pathways of various tissues implicated in the pathogenesis of nonalcoholic steatohepatitis, such as liver and adipose tissue [5]. Leptin is an anorexigenic and pro-inflammatory adipokine that links energy homeostasis to immune system activity [6,7]. The pleiotropic function of leptin is mediated by its binding to leptin receptors in different immune cell types [8]. Due to its role in regulating both arms of the immune response [9], leptin strongly influences inflammation and autoimmune-related disorders [10]. In the context of NAFLD, leptin has potential dual activity in exerting an early protective anti-steatotic response as well as late pro-inflammatory and pro-fibrogenic effects [11]. Leptin-induced oxidative stress and inflammation mediated by Kupffer cells promote the progression of nonalcoholic steatohepatitis [12]; additionally, reports have indicated that oxidative stress is the source of humoral and cellular immunological mechanisms that may contribute to NAFLD progression [13].

Increase in the plasma free fatty acid concentration in healthy individuals were associated with the induction of pro-inflammatory changes and oxidative stress in circulating mononuclear cells [14]. NAFLD progression in humans is characterized by systemic changes in lipid metabolism involving most hepatic and circulating lipids, particularly alterations in the production of n-6 polyunsaturated fatty acids [15, 16]. It was established that ox-NASH, a risk score that incorporates the plasma concentration of linoleic acid and one of its oxidation products, correlates with the primary altered histological features and with the severity of NAFLD [17, 18]. Thus, the inflammatory response of mononuclear cells exposed to linoleic acid is potentially associated with NAFLD progression.

Curcumin, a pigment extracted from the rhizomes of *Curcuma longa*, has strong antioxidant, anti-inflammatory and anti-cancer properties. Curcumin acts by either directly interacting with numerous molecular targets or altering gene expression and signaling pathways [19]. It can prevent obesity and obesity-related chronic diseases [20, 21] and has anti-inflammatory properties on monocytes and macrophages [22] as well as anti-proliferative effects on T cells [23].

Under the hypothesis that a prevailing inflammatory state in NAFLD would be amplified by linoleic acid and leptin, we aimed to evaluate the effects of these molecules on the production of cytokines and reactive oxygen species in immune cells from patients with NAFLD. Given that curcumin may protect against linoleic acid- and leptin-induced inflammatory stimuli, we assessed the ex vivo modulatory effects of curcumin on human immune cells and its potential protective effects against NAFLD progression in a high-fat diet (HFD)-induced NAFLD/nonalcoholic steatohepatitis mouse model.

## Materials and methods

### Ex vivo studies in human NAFLD

#### Patients and general procedures

Blood samples and liver biopsies were collected from 72 adult patients with confirmed NAFLD at the Gastroenterology División of the José de San Martin (JSM) Clinical Hospital (Table 1). The experimental protocols and sample studies were approved by the Ethics Committee of the JSM Clinical Hospital at the University of Buenos Aires and followed the internationally endorsed standards as stated in the Declaration of Helsinki. All subjects were informed of the aim of the study and gave their written informed consent. Accordingly, we collected no more than 15 ml of blood from each patient for this project. Unfortunately, the blood sample volume was insufficient to perform each assay in every subject, and the amount of liver biopsy tissue was inadequate for more than one assay per sample.

**Table 1:** Demographic, anthropometric and biochemical baseline data of patients with NAFLD and control individuals.

Variable (unit)	Control group #1	Control group #2	NAFLD
Gender	32 M/12 F	10 M/4 F	22 M/50 F
Age(years)	50.50±4.90	56.00±6.11	59.50±4.17
Body mass index(kg/m^2^)	23.90±1.06	24.31±3.10	32.41±1.52*
Waist Circumference (cm)	84.00±5.33	86.00±4.43	105.4±5.14**
HOMA-IR	1.31±0.32	ND	3.22±0.33**
Basal glucose (mg/dL)	93.50±1.10	93.60±2.91	95.83±6.99
Insulin (μU/mL)	5.43±0.62	ND	17.54±2.73
Total cholesterol (mg/dL)	174.50±4.12	170.00±2.22	198.8±13.30
Triglycerides(mg/dL)	111.00±3.01	109.01±4.30	165.8±25.70
Aspartate aminotransferases (IU/L)	24.00±0.62	ND	40.43±7.92
Alanineaminotransferases (IU/L)	25.00±0.71	ND	49.00±12.70

The individuals in control group #1 were recruited among staff members at the Institute of Immunology, Genetics and Metabolism at JSM Clinical Hospital. We obtained biopsies from a second control group (control group #2) consisting of 14 individuals who underwent anti-reflux surgery or cholecystectomy at the Gastroenterology División of the JSM Clinical Hospital. Patients with NAFLD were recruited at the Gastroenterology División of the José de San Martin (JSM) Clinical Hospital. Abbreviations: M, males; F, females; ND: not determined. A body mass index between 25 and 29.9 was considered overweight, whereas a value greater than 30 was considered obese. Fasting glucose and insulin were measured using commercial kits. HOMA-IR, homeostatic assessment model for insulin resistance. HOMA-IR was calculated using the following formula with fasting values: HOMA-IR = [glucose (mg/dL) * insulin (μU/mL)/405]. The reference ranges for total cholesterol and triglycerides are 150 to 199 mg/dL and 31 to 119 mg/dL, respectively. The normal alanine and aspartate aminotransferase levels are ≤32 and ≤48 IU/L, respectively. The data are presented as the mean values ± SEM.

* p< 0.05,

** p< 0.01.

A diagnosis of NAFLD was based on daily alcohol consumption (<20 g/day), the absence of other causes of liver disease, and scoring of macrovesicular steatosis, lobular inflammation and ballooning degeneration [24].

Blood from 44 age-matched and metabolically healthy control individuals were recruited among staff members at the Institute of Immunology, Genetics and Metabolism at JSM Clinical Hospital. All the staff was given information regarding the study, and those who intended to participate were told to contact the investigators. None of the control subjects used medications within 6 months previous to the study, consumed more than 20 g/day of alcohol, or had a body mass index higher than 25 kg/m^2^. Additionally, we obtained biopsies from a second control group comprising 14 individuals who underwent either anti-reflux surgery or cholecystectomy at the Gastroenterology División of the JSM Clinical Hospital (Table 1, control groups #1 and #2). Liver biopsies from control group #2 were included to compare the infiltrating cell subpopulations in the liver and the Th1 gene profile expression with those of patients with NAFLD.

Fasting glucose and insulin levels were measured using commercially available kits. The homeostatic assessment model for insulin resistance (HOMA-IR) was calculated using the following formula with fasting values: HOMA-IR = [glucose (mg/dL) * insulin (μU/mL)/405]. The serum levels of total cholesterol, triglycerides, and the aspartate and alanine aminotransferases were determined with a Cobas C-501 autoanalyzer (Roche Diagnostics, Mannheim, Germany) using standardized methods.

#### Cell isolation

Peripheral blood mononuclear cells (PBMCs) were obtained using Ficoll-Hypaque (Pharmacia Biotech, Uppsala, Sweden) density gradient centrifugation and resuspended in Roswell Park Memorial Institute (RPMI) 1640 medium (Sigma Chemical Co., Saint Louis, MO, USA) supplemented with 10% fetal bovine serum, 2 mmol/L L-glutamine, and 50 μg/mL gentamicin.

Liver tissue samples from human biopsies were incubated for 20 min at 37°C in Hank´s balanced salt solution containing 0.05% collagenase and filtered through a 70-μm mesh filter. Liver cell suspensions were washed twice in phosphate-buffered saline, centrifugated at 2000 rpm for 5 min and resuspended in supplemented RPMI 1640.

#### Intracellular reactive oxygen species production

**Fig 1:**
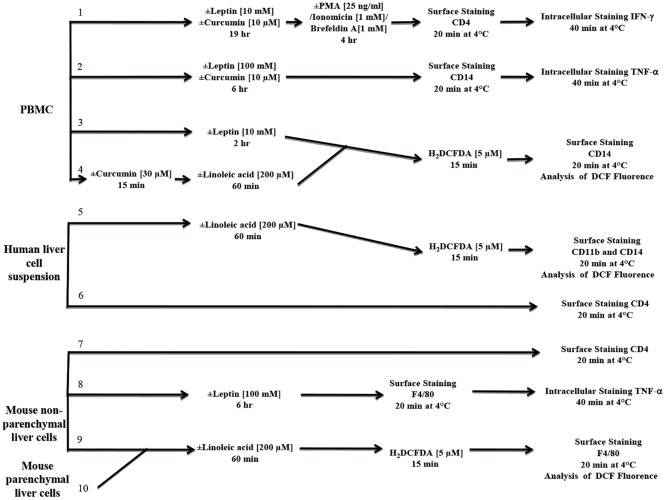
Diagram of the experimental design. Experimental design using human peripheral blood mononuclear cells (PBMCs), human or mouse liver cells. PMA: phorbol myristate acetate, H_2_DCFDA: 2’7’-dichlorofluorescein diacetate.

To evaluate the production of reactive oxygen species, either PBMCs or human liver cell suspensions were incubated at 37°C either in the presence or absence of 200 μM linoleic acid for 60 min or with 10 nM leptin for 2 hr; afterwards, the cells were treated with 5 μM 2’7’-dichlorofluorescein diacetate (H_2_DCFDA) at 37°C for 15 min. Then, cell surface staining was performed using phycoerythrin (PE)-conjugated anti-CD14 monoclonal antibodies (mAbs) (monocytes; Fig 1, pathways 3 and 4) or PE-conjugated anti-CD14 and peridinin-chlorophyll (PerCP)-conjugated anti-CD11b (liver macrophage; Fig 1, pathway 5) mAbs.

The mean 2',7'-dichlorofluorescein (DCF) fluorescence intensity was analyzed either in monocytes identified by their respective forward and side scatter or in human liver macrophages identified by the co-expression of CD14 and CD11b. Data acquisition and analysis were performed on a FACSCalibur flow cytometer (BD, Becton, Dickinson and Company, NJ, USA) using FlowJo software (version 5.7.2). A stimulation index was defined as the ratio between the mean fluorescence intensity of DCF in stimulated and non-stimulated monocytes or macrophages.

#### Liver infiltrating CD4^+^ cells

To evaluate the percentage of CD4^+^ cells infiltrating the liver, human liver cell suspensions were stained with the PerCP-conjugated anti-CD4 mAb at 4°C for 20 min **(**Fig 1, pathway 6). Cells were gated based on side scatter and CD4 expression.

#### Intracellular cytokine production

To evaluate the production of intracellular interferon-gamma (IFN-γ) by T cells, PBMCs were incubated in the presence or absence of 10 nM leptin for 19 hr at 37°C and re-stimulated for 4 hr at 37°C with 25 ng/mL phorbol myristate acetate (PMA) and 1 mM ionomycin in the presence of 1 mM Brefeldin A (Fig 1, pathway 1). To evaluate the production of tumor necrosis factor-α (TNF-α) by monocytes, PBMCs were stimulated in the presence of 100 nM leptin for 6 hr (Fig 1, pathway 2). Cell surface staining was performed using anti-CD4 or anti-CD14 mAbs at 4°C for 20 min as indicated. After the cells were fixed at 4°C for 20 min, PE-conjugated anti-IFN-γ, anti-TNF-α or the corresponding isotype controls were added to the suspensions, and the cells were incubated in 50 μL of permeabilization buffer at 4°C for 40 min. Gates were set on either lymphocytes or monocytes based on their respective forward and side scatter properties and were used to analyze cells stained with either fluorochrome-conjugated control isotypes or specific mAbs. To analyze the peripheral lymphocytes, cells were gated based on the side scatter and CD4 expression, and IFN-γ expression was measured in the selected CD4^+^ population. For monocyte analysis, cells were gated in a forward vs. side scatter dot plot (region 1). Within region 1, CD14^+^ cells were further gated in a side scatter vs. FL2 dot plot (region 2), and TNF-α production was analyzed in the region 2 population. Data acquisition and analysis were performed using flow cytometry as described above.

To quantify the effect of leptin on intracellular IFN-γ and TNF-α production, we calculated the fold of increase index as the ratio between the percentages of either CD4^+^IFN-γ^+^ or CD14^+^TNF-α^+^ cells among the leptin-stimulated and non-stimulated cell populations.

#### Quantitative polymerase chain reaction

Liver tissue was preserved in TRIZOL reagent (GIBCO BRL, LifeTechnologies Inc., Grand Island, NY, USA) and homogenized using Omni Tips (OMNI International, NW, Kennesaw, GA, USA). Total RNA was isolated according to the manufacturer´s protocol and reverse transcribed using a two-step method with SuperScriptII First-Strand Synthesis System (Invitrogen, Life Technologies, Carlsbad, CA, USA) with oligo (dT) primers. The forward and reverse primers were as follows: IFN-γ 5´-CTGTTTTAGCTGCTGGCGAC-3´ and 5´-GAGTGTGGAGACCATCAAGGA-3´; CCL20, 5´-AGCATTGATGTCACAGCCTTC-3´ and 5´-TCAGTGCTGCTACTCCACCT-3´; T-box transcription factor (T-bet), 5´-ATTGACAGTTGGGTCCAGGC-3´ and 5´-TGACTGCCTACCAGAATGCC-3´; and glyceraldehyde 3-phosphate dehydrogenase (GADPH), 5´-cgaccactttgtcaagctca-3´ and 5´-acatggcctccaaggagtaa-3´. GADPH was used as an internal control. Quantitative polymerase chain reaction (qPCR) was performed on a Stratagene Mx3005p RT-PCR Detection System (Agilent Technologies, La Jolla, CA, USA) using SYBR Green PCR Master Mix (applied Biosystems, Life Technologies, Carlsbad, CA, USA). Relative gene expression was calculated using the 2^-ΔΔCt^ method.

#### Modulatory effects of curcumin on PBMCs

PBMCs were pre-incubated with 30 μM curcumin at 37°C for 15 min prior to linoleic acid stimulation and incubation with H_2_DCFDA (Fig 1, pathway 4). To measure IFN-γ and TNF-α production, PBMCs were co-treated with leptin and 10 μM curcumin at 37°C for either 19 hr (Fig 1, pathway 1) or 6 hr (Fig 1, pathway 2).

### In vivo studies in mice

#### Mice, diets and general procedures

Thirty-two inbred male C57BL/6J mice (4 weeks old) were purchased from the School of Veterinary Sciences at the University of Buenos Aires. Of these, 16 mice were used to develop the NAFLD/nonalcoholic steatohepatitis model, and the remaining 16 were designated as corresponding controls. Starting at 5 weeks of age, mice were fed either normal chow (5% Kcal from fat) or a HFD (60% Kcal from fat) (OpenSource Diets, Research Diets, Inc., New Brunswick, NJ, USA) *ad libitum*. After 4 weeks of feeding, mice fed either a HFD or normal chow were randomly divided in the following 4 groups (8 mice per group): (1) HFD, (2) curcumin-treated HFD (HFD+curcumin), (3) normal chow (NC), and (4) curcumin-treated normal chow (NC+curcumin). The curcumin-treated groups received 2 g of curcumin/kg of diet, and the mouse body weight and food consumption were recorded weekly. After 24 weeks of dietary treatments, an intraperitoneal glucose tolerance test was performed, and the mice were euthanized via carbon dioxide anesthesia followed by cervical dislocation in order to collect blood and liver tissue. Due to the limited blood volume obtained from each mouse, the blood was used for biochemical characterization but not for PBMC stimulation. The blood glucose concentration was determined using blood glucose monitors (Accu-Chek Active, Roche Applied Science, Indianapolis, IN, USA) at times 0 and 120 min after an intraperitoneal injection of 2 g/kg glucose. Serum levels of total cholesterol as well as of the aspartate and alanine aminotransferases were determined as indicated in the "Patients and General Procedures" subsection. Hematoxylin & eosin staining was performed for NAFLD activity score calculations, which are based on the presence of macrovesicular steatosis, lobular inflammation and ballooning degeneration. Moreover, the sections were scored for hepatic fibrosis as follows: 0, intact liver with no foci; 1, perisinusoidal or periportal fibrosis; 2, perisinusoidal and periportal fibrosis; 3, septal or bridging fibrosis; and 4, cirrhosis and regenerative nodule formation. Masson´s trichrome staining was conducted to confirm the presence of fibrosis.

Animals were handled in accordance with the principles of the Guide for the Care and Use of Laboratory Animals established by the National Institutes of Health. Experimental protocols were approved by the Institutional Committee for Use and Care of Laboratory Animals (CICUAL, School of Medicine of the University of Buenos Aires, Argentina).

Accordingly, we used the minimum number of live animals necessary to achieve our goals, and all efforts were made to minimize pain and distress during animal husbandry and experimental assessments.

#### Liver cell isolation

Enzymatic digestion of mouse liver tissue was performed as described above. The resulting liver cell suspensions were centrifuged at 400 rpm for 5 min to separate parenchymal cells (i.e., hepatocytes) from non-parenchymal cells. The non-parenchymal cell-enriched pellets were washed twice in phosphate-buffered saline at 2000 rpm for 5 min and resuspended in RPMI 1640 medium.

#### Intracellular reactive oxygen species production

To evaluate the production of reactive oxygen species, the non-parenchymal and parenchymal fractions from digested mouse livers were separately stimulated at 37°C for 60 min in the presence or absence of 200 μM linoleic acid followed by a 15-min incubation with 5 μM H_2_DCFDA at 37°C (Fig 1, pathways 9 and 10). Then, cell surface staining in the non-parenchymal fraction was performed by incubating the cells with PerCP-conjugated anti-F4/80 mAbs at 4°C for 20 min. DCF fluorescence was analyzed in liver macrophages identified by the expression of the F4/80 antigen. Data acquisition and analysis were performed using flow cytometry as described above. The stimulation index was defined as the ratio between the mean DCF fluorescence intensity in macrophages (non-parenchymal fraction) or hepatocytes (parenchymal fraction) that were stimulated or resting.

#### Intracellular cytokine production

To evaluate TNF-α production in liver macrophages, cells were incubated for 6 hr at 37°C in presence or absence of 100 nM leptin. Cell surface staining was then performed using a PerCP-conjugated anti-F4/80 mAb at 4°C for 20 min (Fig 1, pathway 8). After the cells were fixed at 4°C for 20 min, either PE-conjugated anti-TNF-α or the corresponding isotype control Ab was added, and cells were incubated in 50 μL of permeabilization buffer at 4°C for 40 min. Data acquisition and analysis were performed as described above. Within the resulting side scatter vs. FL3 dot plot, a gate was set for F4/80^+^ cells. To quantify the effect of leptin on intracellular TNF-α production, the fold of increase index was calculated as the ratio between the percentages of F4/80^+^TNF-α^+^ cells in leptin-stimulated and non-stimulated cells.

#### Liver infiltrating CD4^+^ cells

Mouse non-parenchymal cell suspensions were stained with PerCP-conjugated anti-CD4 mAb at 4°C for 20 min (Fig 1, pathway 7), and cells were gated based on the side scatter and CD4 expression.

#### Statistical analysis

GraphPad Prism software (GraphPad, San Diego, CA, USA) was used for all the analyses. We performed either two-tailed Mann-Whitney U tests or Wilcoxon signed rank tests to compare data between groups. The Kruskal-Wallis test was used to compare data among three or more groups. For all the experiments, the values are expressed as the median and 25^th^-75^th^ interquartile ranges. Spearman's rank correlation coefficients were used to test the correlation between parameters.

## Results

### Ex vivo studies in human NAFLD

#### Effect of linoleic acid on reactive oxygen species production in monocytes and liver macrophages

**Fig 2:**
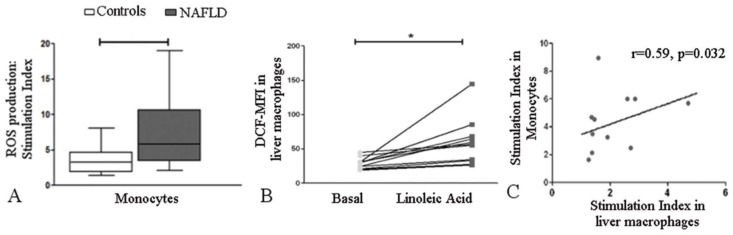
Effect of linoleic acid on reactive oxygen species production in human monocytes and liver macrophages. (A) The stimulation index for reactive oxygen species production in monocytes was higher in patients with NAFLD (n = 12) than in control subjects (n = 10). The box and whiskers indicate the non-parametric statistics: the median, lower and upper quartiles and confidence interval around the median. A two-tailed Mann-Whitney U test was used; *p = 0.036. (B) DCF-MFI, 2', 7’-dichlorofluorescein median fluorescence intensity. Linoleic acid increased reactive oxygen species production in liver macrophages from patients with NAFLD (n = 12). Lines connect the “Basal” and “Linoleic acid” values for each patient. A Wilcoxon matched-pairs signed rank test was performed; *p = 0.001. (C) The stimulation index in monocytes and liver macrophages from patients with NAFLD were positively correlated. Spearman´s rank correlation coefficients test was used.

After cells were stimulated with linoleic acid, the stimulation index was significantly increased in monocytes from patients with NAFLD than in monocytes from control subjects [5.86 (3.43–10.67) vs. 3.24 (1.87–4.70), p = 0.036] (Fig 2A). Linoleic acid also induced reactive oxygen species production in liver macrophages from patients with NAFLD (p = 0.001) (Fig 2B). A positive correlation between linoleic acid-induced reactive oxygen species generation in monocytes and reactive oxygen species generation in liver macrophages was observed in patients with NAFLD (r = 0.59, p = 0.032) (Fig 2C).

#### Effect of leptin on TNF-α and reactive oxygen species production in monocytes

**Fig 3:**
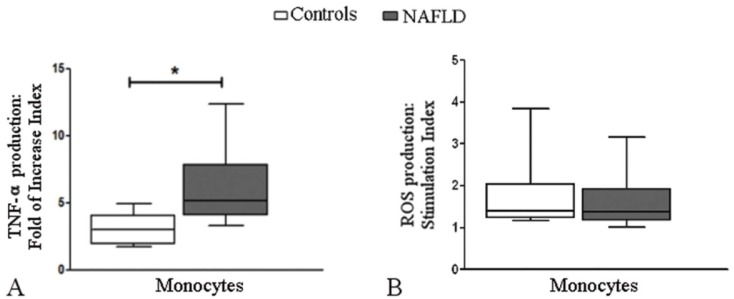
Effect of leptin on TNF-α and reactive oxygen species production in human monocytes. (A) The fold of increase index for TNF-α production was higher in monocytes from patients with NAFLD (n = 10) than those from control subjects (n = 10); however, when monocytes were stimulated with leptin, the stimulation index for reactive oxygen species production (B) was similar in patients with NAFLD (n = 10) and control subjects (n = 10). The box and whiskers indicate the non-parametric statistics: median, lower and upper quartiles and confidence interval around the median. A two-tailed Mann-Whitney U test was used, *p = 0.004.

Leptin stimulated TNF-αα production in peripheral monocytes from control subjects and patients with NAFLD; however, compared to the control subjects, the patients with NAFLD presented higher fold of increase indexes [2.99 (1.91–4.03) vs. 5.04 (3.87–8.54), respectively; p = 0.004] (Fig 3A). Similar indexes of reactive oxygen species production were observed in monocytes from both groups [NAFLD: 1.34 (1.16–1.93), control: 1.40 (1.22–2.03), p = 0.437] (Fig 3B).

#### Effect of leptin on IFN-γ production in CD4^+^ cells

**Fig 4:**
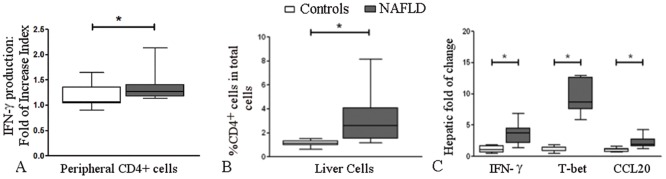
The effects of leptin on IFN-γ production and T cell-associated alterations in liver samples from patients with NAFLD. (A) The fold of increase index for IFN-γ production in leptin-stimulated circulating CD4^+^ cells was higher in patients with NAFLD (n = 10) than in control subjects (n = 10; *p = 0.011). **(B)** The percentage of CD4^+^ cells among the total non-parenchymal cell population was higher in patients with NAFLD (n = 10) than in control subjects (n = 10), *p = 0.030. **(C)** Compared with control subjects (n = 9), patients with NAFLD (n = 9) showed increased hepatic mRNA expression levels of IFN-γ (*p = 0.012), T-bet (*p = 0.020) and CCL20 (*p = 0.007) as measured by quantitative PCR. The 2^-ΔΔCt^ method was used to calculate the mRNA fold change. The box and whiskers indicate the non-parametric statistics: the median, lower and upper quartiles and confidence interval around the median. A two-tailed Mann-Whitney U test was used for the statistical analysis.

Leptin stimulated IFN-γ production in circulating CD4^+^ cells from control subjects and patients with NAFLD; however, we observed higher fold of increase indexes in patients with NAFLD than in control subjects [1.26 (1.17–1.40) vs. 1.06 (1.04–1.35), respectively; p = 0.011] (Fig 4A).

#### Infiltrating CD4^+^ cells into the liver

When intrahepatic lymphocyte subpopulations were compared between patients with NAFLD and control subjects, the percentage of CD4^+^ cells among the total cell population was higher in patients with NAFLD [2.81 (1.83–4.11) vs. 1.12 (0.70–1.25), respectively; p = 0.030] (Fig 4B).

#### Gene expression in liver biopsies

Compared to samples from control subjects, liver biopsies from patients with NAFLD showed increased expression levels of IFN-γ [3.73 (2.17–4.5) vs. 1.05 (0.61–1.7), p = 0.012], T-bet [8.67 (7.53–12.66) vs. 0.96 (0.86–1.42), p = 0.020] and CCL20 [1.89 (1.62–2.77) vs. 1.12 (0.73–1.23), p = 0.007] (Fig 4C).

#### The reversal effects of curcumin

**Fig 5:**
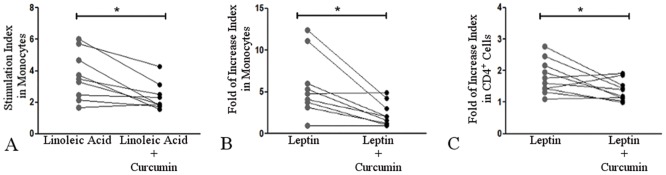
The reversal effects of curcumin on peripheral immunological cells. Ex vivo curcumin treatment of PBMCs from patients with NAFLD resulted in decreases in **(A)** linoleic acid-induced reactive oxygen species generation (n = 9, *p = 0.011) and **(B)** leptin-induced TNF-α production (n = 9, *p = 0.016) by monocytes. **(C)** Ex vivo curcumin treatment of PBMCs from patients with NAFLD resulted in decreased IFN-γ production in CD4^+^ cells (n = 9, *p = 0.048). Lines connect the “Linoleic acid” and “Linoleic acid+Curcumin” stimulation indexes or the “Leptin” and "Leptin+Curcumin" fold of increase indexes for each patient. A Wilcoxon matched-pairs signed rank test was performed.

An ex vivo analysis of the effects of curcumin on PBMCs showed a decrease in linoleic acid-induced reactive oxygen species generation (p = 0.011) (Fig 5A) and leptin-induced TNF-α production (p = 0.016) (Fig 5B) in monocytes from patients with NAFLD. Curcumin lowered these parameters to levels comparable to those in cells from control subjects (data not shown). Interestingly, curcumin also ameliorated leptin-induced IFN-γ production in CD4^+^ cells (p = 0.048) (Fig 5C).

### In vivo studies in mice

#### HFD-induced metabolic and liver histological alterations and the reversal effects of curcumin

**Fig 6:**
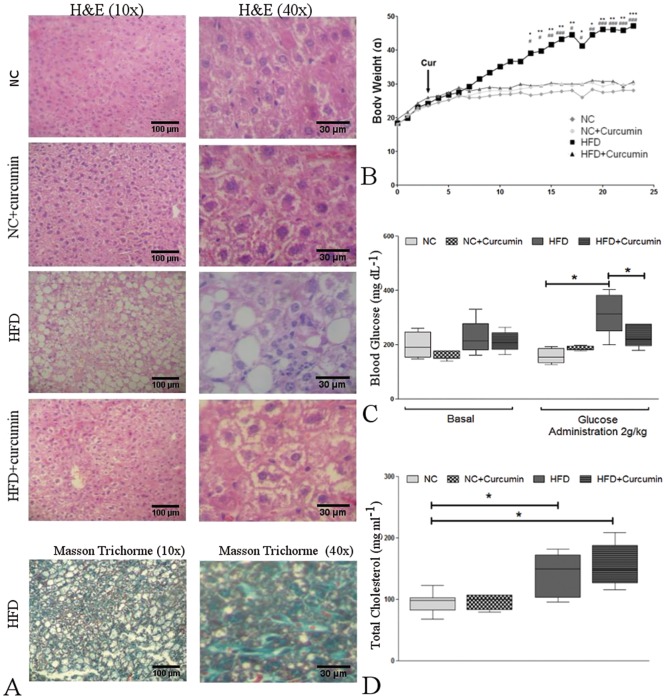
HFD-induced metabolic and histological alterations in the liver and the reversal effects of curcumin. **(A)** Top: Hematoxylin and eosin staining of liver sections from normal chow (NC)-fed and high-fat diet (HFD)-fed mice with or without curcumin supplementation (10X and 40X magnification). Curcumin prevented HFD-induced steatosis, ballooning and liver inflammation. Bottom: Masson's trichrome staining of liver sections from HFD-fed mice reveals signs of mild perisinusoidal fibrosis (10X and 40X magnification). **(B)** Body weight gain in NC- and HFD-fed mice with or without curcumin supplementation, starting at week 4 (arrow) (*p<0.05, **p<0.01 and ***p<0.001, HFD vs. NC). Curcumin prevented HFD-induced weight gain (#p<0.05, ##p<0.01 and ###p<0.001, HFD vs. HFD+curcumin). **(C)** The blood glucose concentrations were measured upon conclusion of the dietary treatments, and glycemia was determined at basal conditions (Basal) and after glucose administration. Left: a similar blood glucose concentration was observed among the four groups. Right: hyperglycemia was observed in HFD-fed mice at 120 min after an intraperitoneal glucose injection (2 g/kg). Curcumin ameliorated the hyperglycemic conditions. (*p<0.01). **(D)** Total serum cholesterol levels were measured upon conclusion of the dietary treatments. The HFD induced higher levels of cholesterol than the normal chow regardless of curcumin administration (*p<0.05). The box and whiskers show the non-parametric statistics: the median, lower and upper quartiles and confidence interval around the median. The Kruskal-Wallis test with Dunn’s post-test was performed.

Compared to normal chow-fed mice, HFD mice exhibited enhanced weight gain from week 14 to the end of the dietary treatment [40.80 (36.20–47.50) g vs. 30.90 (29.55–33.35) g, respectively; p<0.001], (Fig 6B) and had higher serum glucose concentrations at 120 min after a glucose injection [313 (249–382) mg/dl vs. 155 (131.8–185) mg/dl, p<0.01] (Fig 6C) at the end of the dietary treatment. Higher total cholesterol levels were also observed in the HFD [150 (103–172) mg/dl, p<0.05] and HFD+curcumin [149(126.5–188) mg/dl, p<0.05] groups than in the normal chow-fed mice [98 (82–102.5) mg/dl] (Fig 6D). However, the levels of triglycerides as well as of the aspartate and alanine aminotransferases were similar among all the groups (data not shown).

Hematoxylin & eosin staining revealed steatohepatitis with macrovesicular and microvesicular liver steatosis, scattered foci of lobular inflammation and ballooning degeneration in the livers from HFD-fed mice (Fig 6A). Hematoxylin & eosin scoring and Masson's trichrome staining revealed that 70% of the HFD-fed mice exhibited signs of mild perisinusoidal fibrosis. In contrast, none of the NC mice developed fibrosis.

Compared to the non-supplemented diets, in vivo curcumin supplementation prevented weight gain [30.60 (30.45–33.05) g] among the HFD group (p<0.05) (Fig 6B) and improved glucose elimination from the blood of HFD mice [HFD+curcumin: 219 (194.5–276) mg/dl, p<0.05, vs. HFD] (Fig 6C). Curcumin administration also prevented liver injury by reducing steatosis, inflammation and ballooning degeneration (Fig 6A). Furthermore, only 20% of HFD mice supplemented with curcumin exhibited signs of mild perisinusoidal fibrosis.

#### Effect of linoleic acid on reactive oxygen species production in liver cells and the reversal effects of curcumin

**Fig 7:**
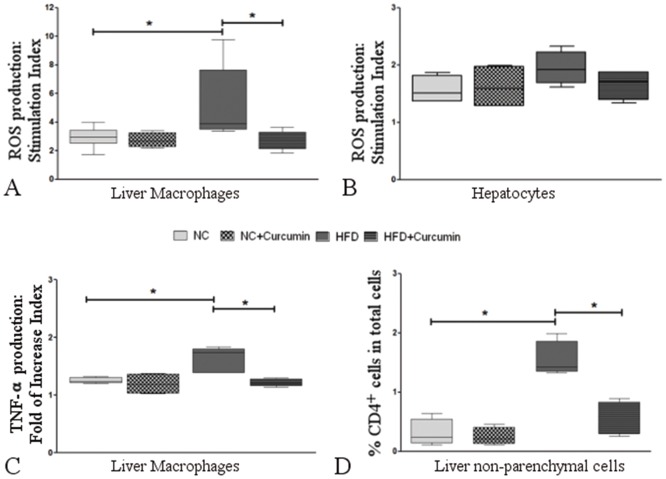
Curcumin effects on linoleic acid- and leptin-induced the production of reactive oxygen species and cytokines as well as the infiltration of CD4^+^ cells in HFD-fed mice. **(A)** After they were treated with linoleic acid ex vivo, liver macrophages from HFD-fed mice showed a higher stimulation index for reactive oxygen species production (*p<0.05 vs. normal chow-fed mice). In vivo curcumin administration of HFD-fed mice (HFD+curcumin) prevented the increase in the stimulation index (*p<0.05 vs. HFD-fed mice). **(B)** Ex vivo linoleic acid stimulation of hepatocytes from all the experimental groups resulted in similar stimulation indexes for reactive oxygen species production. **(C)** TNF-α production induced by ex vivo leptin treatment was higher in liver macrophages from HFD-fed mice (*p<0.05 vs. normal chow-fed mice). In vivo curcumin treatment of HFD-fed mice also prevented the increase in TNF- α production (*p<0.05, HFD+curcumin vs. HFD). **(D)** The percentage of CD4^+^ cells among the non-parenchymal cell populations was higher in HFD-fed mice (*p<0.01 vs. normal chow-fed mice). In vivo curcumin treatment also prevented the increase in CD4^+^ cell recruitment (*p<0.01, HFD+curcumin vs. HFD). The box and whiskers show the non-parametric statistics: the median, lower and upper quartiles and confidence interval around the median. The Kruskal-Wallis test with Dunn’s post-test was performed.

Upon stimulation with linoleic acid, liver macrophages from HFD-fed mice showed a higher production of reactive oxygen species than macrophages from NC mice [3.88 (3.46–7.63) vs. 2.95 (2.48–3.42) p<0.05] (Fig 7A). However, liver macrophages treated with curcumin showed a reduction in the linoleic acid-induced increase in reactive oxygen species production [2.49 (2.12–3.30), p<0.05, Fig 7A]. Linoleic acid stimulation of hepatocytes from all the experimental groups resulted in similar stimulation indexes for reactive oxygen species production (Fig 7B).

#### Effects of leptin on TNF-α production in liver macrophages and the reversal effect of curcumin

When stimulated with leptin, liver macrophages from HFD-fed mice showed a more profound increase in TNF-α production than NC mice [1.73 (1.38–1.80) vs. 1.24 (1.21–1.30), p<0.05]. Curcumin prevented the leptin-induced increase in TNF-α production [1.21(1.15–1.28), p<0.05] in liver macrophages (Fig 7C).

#### Infiltrating CD4^+^ cells in the liver and the reversal effect of curcumin

Flow cytometry of non-parenchymal cells in the liver showed that the percentage of CD4^+^ cells was higher in HFD-fed mice than in normal chow-fed mice [1.42 (1.34–1.85) vs. 0.24 (0.13–0.54), p<0.05]. However, the recruitment of CD4^+^ cells into the liver was attenuated in curcumin-treated HFD-fed mice [0.52 (0.29–0.83), p<0.01, HFD+curcumin vs. HFD] (Fig 7D).

## Discussion

Our present findings concerning human NAFLD indicate that linoleic acid exacerbated reactive oxygen species generation in monocytes and liver macrophages and that leptin promoted increased TNFα production in monocytes coupled with more CD4^+^ cell differentiation into Th1 cells. Ex vivo curcumin treatment of PBMCs from patients with NAFLD showed a reversion of the hyperresponsiveness to linoleic acid and leptin observed in monocytes and CD4^+^ cells. The response of these immune cells may reveal higher baseline activity of pro-inflammatory enzymes and signaling pathways within these cells. For example, linoleic acid-induced acceleration of nicotinamide adenine dinucleotide phosphate (NADPH) oxidase assembly and/or co-activation of protein kinase C [25] and leptin-induced activation of receptor-associated kinases, insulin receptor substrate-1 and associated signaling pathways [26] might contribute to the observed increases in reactive oxygen species, TNF-α production and Th1 differentiation. We observed a positive correlation between linoleic acid-induced reactive oxygen species generation in monocytes and the number of liver macrophages from patients with NAFLD. Although this correlation does not directly add a predictive value to peripheral linoleic acid-induced reactive oxygen species production, this observation warrants further investigation.

Curcumin exerts an ex vivo protective effect against linoleic acid- and leptin-induced inflammatory stimuli which may be mediated by downregulating the expression of transcription factors and reducing the activity of associated signaling pathways [19].

The analysis of gene expression and intrahepatic accumulation of CD4^+^ cells was addressed to evaluate T cell-related alterations in the liver. T-bet is a critical regulator of the expression of chemokines and their corresponding receptors and induces Th1 differentiation of CD4^+^ lymphocytes [27]. Lymphocyte recruitment is an essential process associated with NAFLD progression, and enhanced infiltration of CD4^+^ cells coupled with elevated expression levels of T-bet, IFN-γ and CCL-20 in liver biopsies indicate a prevailing inflammatory state in patients with NAFLD.

The in vivo approach of this study involved the development of an experimental model that reproduced the primary characteristics of nonalcoholic steatohepatitis in humans. Mice fed a HFD developed steatohepatitis with mild fibrosis, exhibited enhanced weight gain, and presented higher total cholesterol levels and poor elimination of glucose from the blood. In contrast, despite a great number of studies describing elevated hepatic transaminase levels in HFD-induced experimental NAFLD, we did not observe increased levels of either aspartate or alanine aminotransferases. However, our results were in agreement with a report that observed hepatic lipid accumulation and mild fibrosis with no changes in the aspartate and alanine aminotransferase levels in rats fed a HFD [28]. We also performed experiments with our mouse model that corresponded to most of the human studies. As in the livers from patients with NAFLD, enhanced reactive oxygen species generation and TNF-α production by mouse liver macrophages revealed an overall hyperactive state in the HFD-fed group. This condition may result from the diet-induced elevation of the intrahepatic levels of pro-inflammatory cytokines and glucose, which might promote the activation of NADPH oxidase [29] and the hyperresponsiveness to pro-inflammatory mediators such as linoleic acid and leptin. Reactive oxygen species production by linoleic acid-stimulated hepatocytes is probably associated with the high intrinsic antioxidant capacity of this cell type [30]. As in human NAFLD, increased intrahepatic accumulation of CD4^+^ cells was found to be an immunological alteration in HFD-fed mice.

In vivo curcumin administration interfered with the development of distinctive characteristics associated with the NAFLD/nonalcoholic steatohepatitis phenotype, such as obesity and glucose intolerance, and improved histological alterations, including fibrosis and the intrahepatic accumulation of CD4^+^ cells. The overall beneficial effects of curcumin on the development of HFD-induced nonalcoholic steatohepatitis may result from its direct interaction with different metabolically active tissues [31]. However, because HFD-fed mice developed obesity and glucose intolerance and these metabolic alterations influence liver injury and nonalcoholic steatohepatitis-associated fibrosis [32], we cannot disregard the possibility that local histological improvement may be an effect secondary to the systemic properties of curcumin. The lack of an exacerbated response of liver macrophages from the HFD+curcumin group to pro-inflammatory mediators may result from the effects of curcumin on multiple targets during in vivo mouse treatments [19].

An important strength of this study is the similarities between the developed animal model and the primary general features of human nonalcoholic steatohepatitis. Nevertheless, we recognize several limitations of our study. First, we used supraphysiological concentrations of leptin and linoleic acid in the ex vivo human experiments [33, 34]. Second, we used PBMCs and liver cell suspensions rather than isolated monocytes, T cells and macrophages. Third, the restrictions imposed by the limited blood volume and the small size of percutaneous liver biopsies resulted in insufficient samples to perform each assay for every subject. Regarding control livers, we could only utilize biopsies that were removed from patients who required a diagnostic test; these biopsies were performed to rule out other diagnoses, and the samples ended up being control livers. Thus, another limitation was the bias that could have been established in selecting patients who underwent anti-reflux surgery or cholecystectomy as the source of control livers.

Based on a post hoc analysis using G*Power 3.1.9.2 (Universität Düsseldorf, Germany), the power of the statistically significant results from human studies fell within the acceptable range of 0.551 to 1.000 (S1 Table). In the case of the leptin-induced effect on CD4^+^ circulating cells (which was calculated with the lowest statistical power), we have used our results as preliminary data and calculated that 2–3 times more patients are needed to reach a value of at least 0.800.

## Conclusions

We have demonstrated the remarkable pro-inflammatory and pro-oxidant influence of linoleic acid and leptin in human and mouse NAFLD/nonalcoholic steatohepatitis and showed that curcumin prevented the development of immunological alterations in this disease. A preventive treatment with curcumin might target circulating monocytes and liver macrophages as well as peripheral and hepatic CD4^+^ cells. The translational potential of these results requires additional research.

## Supporting information

S1 Table Statistical power of human studies. The power of the human studies was calculated by a post hoc analysis using G*Power 3.1.9.2 (Universität Düsseldorf, Germain). ROS: oxygen reactive species, TNF-α: tumor necrosis factor-α.
(DOC)

## Abbreviations

CCL20: CC-chemokine ligand 20
DCF: 2';7'-dichlorofluorescein
GAPDH: glyceraldehyde 3-phosphate dehydrogenase
H_2_DCFDA: 2’7’-dichlorofluorescein diacetate
HFD: high-fat diet
HOMA-IR: homeostatic model assessment of insulin resistance
IFN-γ: interferon-gamma
mAbs: monoclonal antibodies
NADPH oxidase: nicotinamide adenine dinucleotide phosphate-oxidase
NAFLD: nonalcoholic fatty liver disease
PBMCs: peripheral blood mononuclear cells
PMA: phorbol myristate acetate
qPCR: quantitative polymerase chain reaction
T-bet: T-box transcription factor TBX21
Th1: T helper 1
TNF-α: tumor necrosis factor-α

## References

[1] Tiniakos DG, Vos MB, Brunt EM. Nonalcoholic fatty liver disease: pathology and pathogenesis. Annual review of pathology. 2010;5:145–71. 10.1146/annurev-pathol-121808-102132 20078219

[2] Tilg H, Moschen AR. Evolution of inflammation in nonalcoholic fatty liver disease: The multiple parallel hits hypothesis. Hepatology (Baltimore, Md). 2010;52(5):1836–46. 10.1002/hep.24001 21038418

[3] Meli R, Mattace Raso G, Calignano A. Role of innate immune response in non-alcoholic Fatty liver disease: metabolic complications and therapeutic tools. Frontiers in immunology. 2014;5:177 10.3389/fimmu.2014.00177 24795720 PMC4005965

[4] Braunersreuther V, Viviani GL, Mach F, Montecucco F. Role of cytokines and chemokines in non-alcoholic fatty liver disease. World journal of gastroenterology: WJG. 2012;18(8):727–35. 10.3748/wjg.v18.i8.727 22371632 PMC3286135

[5] Vonghia L, Michielsen P, Francque S. Immunological mechanisms in the pathophysiology of non-alcoholic steatohepatitis. International journal of molecular sciences. 2013;14(10):19867–90. 10.3390/ijms141019867 24084730 PMC3821591

[6] Procaccini C, De Rosa V, Galgani M, Carbone F, Cassano S, Greco D, et al Leptin-induced mTOR activation defines a specific molecular and transcriptional signature controlling CD4^+^ effector T cell responses. Journal of immunology (Baltimore, Md: 1950). 2012;189(6):2941–53. 10.4049/jimmunol.1200935 22904304

[7] Cassano S, Pucino V, La Rocca C, Procaccini C, De Rosa V, Marone G, et al Leptin modulates autophagy in human CD4^+^CD25^-^ conventional T cells. Metabolism: clinical and experimental. 2014;63(10):1272–9. 25060689 10.1016/j.metabol.2014.06.010 PMC4180014

[8] Lam QL, Lu L. Role of leptin in immunity. Cellular & molecular immunology. 2007;4(1):1–13. 17349207

[9] Matarese G, Moschos S, Mantzoros CS. Leptin in immunology. Journal of immunology (Baltimore, Md: 1950). 2005;174(6):3137–42. 10.4049/jimmunol.174.6.3137 15749839

[10] Procaccini C, Pucino V, Mantzoros CS, Matarese G. Leptin in autoimmune diseases. Metabolism: clinical and experimental. 2015;64(1):92–104. 25467840 10.1016/j.metabol.2014.10.014

[11] Polyzos SA, Kountouras J, Mantzoros CS. Leptin in nonalcoholic fatty liver disease: a narrative review. Metabolism: clinical and experimental. 2015;64(1):60–78. 25456097 10.1016/j.metabol.2014.10.012

[12] Chatterjee S, Ganini D, Tokar EJ, Kumar A, Das S, Corbett J, et al Leptin is key to peroxynitrite-mediated oxidative stress and Kupffer cell activation in experimental non-alcoholic steatohepatitis. J Hepatol. 2013;58(4):778–84. 10.1016/j.jhep.2012.11.035 23207144 PMC3596459

[13] Sutti S, Jindal A, Locatelli I, Vacchiano M, Gigliotti L, Bozzola C, et al Adaptive immune responses triggered by oxidative stress contribute to hepatic inflammation in NASH. Hepatology (Baltimore, Md). 2014;59(3):886–97. 10.1002/hep.26749 24115128

[14] Tripathy D, Mohanty P, Dhindsa S, Syed T, Ghanim H, Aljada A, Dandona P. Elevation of free fatty acids induces inflammation and impairs vascular reactivity in healthy subjects. Diabetes. 2003;52(12): 2882–7. 14633847 10.2337/diabetes.52.12.2882

[15] Puri P, Wiest MM, Cheung O, Mirshahi F, Sargeant C, Min HK et al The plasma lipidomic signature of nonalcoholic steatohepatitis. Hepatology. 2009;50(6): 1827–38. 10.1002/hep.23229 19937697 PMC5031239

[16] Puri P, Baillie RA, Wiest MM, Mirshahi F, Choudhury J, Cheung O et al A lipidomic analysis of nonalcoholic fatty liver disease. Hepatology. 2007;46(4): 1081–90. 10.1002/hep.21763 17654743

[17] Feldstein AE, Lopez R, Tamimi TA, Yerian L, Chung YM, Berk M et al Mass spectrometric profiling of oxidized lipid products in human nonalcoholic fatty liver disease and nonalcoholic steatohepatitis. J Lipid Res. 2010;51(10): 3046–54. 10.1194/jlr.M007096 20631297 PMC2936759

[18] Alkhouri N, Berk M, Yerian L, Lopez R, Chung YM, Zhang R et al Oxnash score correlates with histologic features and severity of nonalcoholic fatty liver disease. Dig Dis Sci. 2014;59(7): 1617–24. 10.1007/s10620-014-3031-8 24464211 PMC4279921

[19] Shishodia S. Molecular mechanisms of curcumin action: Gene expression. BioFactors. 2013;39(1): 37–55. 10.1002/biof.1041 22996381

[20] Wang S, Moustaid-Moussa N, Chen L, Mo H, Shastri A, Su R et al Novel insights of dietary polyphenols and obesity. J Nutr Biochem. 2014;25(1): 1–18. 10.1016/j.jnutbio.2013.09.001 24314860 PMC3926750

[21] Shapiro H, Bruck R. Therapeutic potential of curcumin in non-alcoholic steatohepatitis. Nutrition Research Reviews. 2005;18(02): 212–21. 19079906 10.1079/NRR2005106

[22] Abe Y, Hashimoto S, Horie T. Curcumin inhibition of inflammatory cytokine production by human peripheral blood monocytes and alveolar macrophages. Pharmacol Res. 1999;39(1): 41–7. 10.1006/phrs.1998.0404 10051376

[23] Kim G, Jang MS, Son YM, Seo MJ, Ji SY, Han SH et al Curcumin inhibits cd4(+) t cell activation, but augments cd69 expression and tgf-beta1-mediated generation of regulatory t cells at late phase. PLOSone. 2013;8(4): e62300 10.1371/journal.pone.0062300 PMC3637266 23658623

[24] Kleiner DE, Brunt EM. Nonalcoholic fatty liver disease: Pathologic patterns and biopsy evaluation in clinical research. Semin Liver Dis. 2012;32(1): 3–13. 10.1055/s-0032-1306421 22418883

[25] Sellmayer A, Obermeier H, Danesch U, Aepfelbacher M, Weber PC. Arachidonic acid increases activation of nadph oxidase in monocytic u937 cells by accelerated translocation of p47-phox and co-stimulation of protein kinase c. Cell Signal. 1996;8(5): 397–402. 8911691 10.1016/0898-6568(96)00077-0

[26] Sanchez-Margalet V, Martin-Romero C, Santos-Alvarez J, Goberna R, Najib S, Gonzalez-Yanes C. Role of leptin as an immunomodulator of blood mononuclear cells: Mechanisms of action. Clin Exp Immunol. 2003;133(1): 11–9. 10.1046/j.1365-2249.2003.02190.x 12823272 PMC1808745

[27] Lazarevic V, Glimcher LH. T-bet in disease. Nat Immunol. 2011;12(7): 597–606. 10.1038/ni.2059 21685955 PMC6290474

[28] Oner-Iyidogan Y, Tanrikulu-Kucuk S, Seyithanoglu M, Koçak H, Doğru-Abbasoğlu S, Aydin AF et al Effect of curcumin on hepatic heme oxygenase 1 expression in high fat diet fed rats: Is there a triangular relationship? Can J Physiol Pharmacol. 2014;92(10): 805–12. 10.1139/cjpp-2014-0174 25211173

[29] Balteau M, Tajeddine N, de Meester C, Ferté L, Battault S, Ravenstein CM et al Nadph oxidase activation by hyperglycaemia in cardiomyocytes is independent of glucose metabolism but requires sglt1. Cardiovasc Res. 2011;92(2): 237–46. 10.1093/cvr/cvr230 21859816

[30] Baffy G. Kupffer cells in non-alcoholic fatty liver disease: The emerging view. Journal of Hepatology. 51(1): 212–23. 10.1016/j.jhep.2009.03.008 19447517 PMC2694233

[31] Aggarwal BB. Targeting inflammation-induced obesity and metabolic diseases by curcumin and other nutraceuticals. Annu Rev Nutr. 2010;30: 173–99. 10.1146/annurev.nutr.012809.104755 20420526 PMC3144156

[32] Farrell GC, Mridha AR, Yeh MM, Arsov T, Van Rooyen DM, Brooling J et al Strain dependence of diet-induced nash and liver fibrosis in obese mice is linked to diabetes and inflammatory phenotype. Liver Int. 2014;34(7): 1084–93. 10.1111/liv.12335 24107103

[33] Huang XD, Fan Y, Zhang H, Maachi M, Wendum D, Paye F et al Serum leptin and soluble leptin receptor in non-alcoholic fatty liver disease. World J Gastroenterol. 2008;14(18): 2888–93. 10.3748/wjg.14.2888 18473416 PMC2710733

[34] de Almeida IT, Cortez-Pinto H, Fidalgo G, Rodrigues D, Camilo ME. Plasma total and free fatty acids composition in human non-alcoholic steatohepatitis. Clin Nutr. 2002;21(3): 219–23. 12127930 10.1054/clnu.2001.0529

