# Supplementary material for: New evidence for the therapeutic potential of curcumin to treat nonalcoholic fatty liver disease in humans
Source: PLoS One. 2017 Mar 3;12(3):e0172900. doi: 10.1371/journal.pone.0172900 (PMC5336246; doi:10.1371/journal.pone.0172900)
Supplement: S1 Table — The power of the human studies was calculated by a post hoc analysis using G*Power 3.1.9.2 (Universität Düsseldorf, Germain). ROS: oxygen reactive species, TNF-α: tumor necrosis factor-α. (DOC) [file pone.0172900.s001.doc]

**S1 Table. Statistical Power of Human S**tudies

| Study | Statistical Power |
| --- | --- |
| Effects of LNA on ROS  production in monocytes | 0.654 |
| Effect of leptin on TNF-α  production in monocytes | 0.934 |
| Effect of leptin on intracellular IFN-γ  production in circulating CD4^+^ cells | 0.245 |
| Liver infiltrating CD4^+^ cells | 0.551 |
| IFN-γ intrahepatic expression | 0.988 |
| T-bet intrahepatic expression | 1.000 |
| CCL-20 intrahepatic expression | 0.954 |
